# Synthesis of Polyethylene Glycol Diacrylate/Acrylic Acid Nanoparticles as Nanocarriers for the Controlled Delivery of Doxorubicin to Colorectal Cancer Cells

**DOI:** 10.3390/pharmaceutics14030479

**Published:** 2022-02-22

**Authors:** Yin Yin Myat, Tanasait Ngawhirunpat, Theerasak Rojanarata, Praneet Opanasopit, Mark Bradley, Prasopchai Patrojanasophon, Chaiyakarn Pornpitchanarong

**Affiliations:** 1 Pharmaceutical Development of Green Innovations Group (PDGIG), Faculty of Pharmacy, Silpakorn University, Nakhon Pathom 73000, Thailand; myat_y@su.ac.th (Y.Y.M.); ngawhirunpat_t@su.ac.th (T.N.); rojanarata_t@su.ac.th (T.R.); opanasopit_p@su.ac.th (P.O.); patrojanasophon_p@su.ac.th (P.P.); 2 School of Chemistry, University of Edinburgh, David Brewster Road, Edinburgh EH9 3FJ, UK; mark.bradley@ed.ac.uk

**Keywords:** colorectal cancer, doxorubicin, nanoparticles, polyethylene glycol diacrylate, acrylic acid

## Abstract

Doxorubicin (Dox) is known for its potential to deliver desirable anticancer effects against various types of cancer including colorectal cancer. However, the adverse effects are serious. This study aimed to synthesize polyethylene glycol diacrylate (PEGDA)/acrylic acid (AA)-based nanoparticles (PEGDA/AA NPs) for Dox delivery to colorectal cancer cells. The NPs were synthesized using free-radical polymerization reaction using the monomers PEGDA and AA with their physical properties, drug loading and release, biocompatibility, and anticancer effect evaluated. The NPs were spherical with a size of around 230 nm, with a 48% Dox loading efficiency and with loading capacity of 150 µg/mg. Intriguingly, the NPs had the ability to prolong the release of Dox in vitro over 24 h and were non-toxic to intestinal epithelial cells. Dox-loaded PEGDA/AA NPs (Dox-NPs) were able to effectively kill the colorectal cancer cell line (HT-29) with the Dox-NPs accumulating inside the cell and killing the cell through the apoptosis pathway. Overall, the synthesized PEGDA/AA NPs exhibit considerable potential as a drug delivery carrier for colon cancer-directed, staged-release therapy.

## 1. Introduction

Colorectal cancer is the third leading cause of cancer-related mortality in men and women [1]. Treatments such as chemotherapy, surgery, and radiotherapy are key current methods used to treat colorectal cancer. Often, to achieve the desired outcome, two or more treatment modalities are combined [2]. Surgery is the first-line strategy for colorectal cancer treatment, especially if detected at an early stage. However, the disadvantage of such a method is recurrence, which may lead to metastasis [3,4]. Although chemotherapeutic agents have proved useful in cancer treatment, patients experience severe side effects, for example, hair loss, fatigue, nausea and vomiting, constipation or diarrhea, anemia, immunosuppression, and other organ toxicities [5,6]. Doxorubicin (Dox), an anthracycline derivative, has been applied in various curative drug combinations for the treatment of ovarian, breast, bladder, lung, and colon cancers [1,7]. Its mechanism of action is intercalating the DNA base pairs and inhibiting DNA and RNA replication that causes DNA damage and induces cellular apoptosis [8], and it is a very potent and cost-effective compound compared with other therapeutic agents [9].

Nanotechnology can be a strategy to overcome some of the drawbacks of chemotherapeutic compounds, such as improving the efficiency of drugs [10]. Notably, polymeric nanoparticles (NPs) have been shown to have promising properties as a drug carrier, for instance, prolonging drug release and, importantly, enabling specific targeting [11,12,13,14,15,16]. One of the most promising pharmacokinetic properties of NPs as drug carriers is the superior accumulation of drug-loaded NPs in tumors compared to normal healthy tissues [10,17,18], improving efficacy along with minimizing its adverse side effects [5,19]. PEGylation is known to create a stealth property for the NPs, which creates a coating layer for its core to avoid interactions with other components in the blood circulation as well as improving biocompatibility. Moreover, PEGylated nanocarriers can be prolonged in the blood circulation due to the prevention of opsonization and phagocytosis. PEGylation commonly occurs at the most outer layer of the nanocarriers, especially lipid-based nanocarriers e.g., liposomes, niosomes, or those conjugated to the functional groups at the outer surface of polymer-based nanocarriers [20,21,22].

PEGDA is a derivative of polyethylene glycol (PEG), with vinyl end groups that can be used for polymerization that is soluble in water and offers very low toxicity [23]. Acrylic acid is a monomer with good water solubility and biocompatibility [24]. In this study, we focused on the synthesis of polyethylene glycol diacrylate (PEGDA)/acrylic acid (AA) NPs for the delivery of Dox into colorectal cancer cells safely and efficiently. The research proposed stealth NPs with negatively ionizable groups for anticancer drug delivery. The NPs were created using swellable and inert monomer to provide stealth properties upon hydration and facilitate compatibility and extended circulation as well as drug captivation without coating necessity. Moreover, the negatively ionized monomer was selected to form interactions with protonable active compounds, herein, Dox.

## 2. Materials and Methods

### 2.1. Materials

PEGDA (MW = 575), AA, Dox hydrochloride, *N*,*N*′-methylene bisacrylamide (MBA), 2,2′-azobis(2-methylpropionamidine) dihydrochloride (V50), and methylthiazolyldiphenyltetrazolium bromide (MTT) were acquired from Sigma Aldrich (St. Louis, MO, USA). Acetonitrile, methanol (HPLC grade), and ethyl acetate were supplied by Merck & Co. (Darmstadt, Germany). Deuterium oxide (D_2_O) was purchased from Cambridge Isotope Laboratories (Tewksbury, MA, USA). Dulbecco’s modified Eagle’s medium (DMEM), fetal bovine serum (FBS), *L*-glutamine, non-essential amino acids, and penicillin-streptomycin were procured from Gibco BRL (Rockville, MD, USA). Annexin V binding buffer, annexin V Alexa Fluor™ 647 conjugate, and SYTOX™ Green nucleic acid stain were bought from Invitrogen (Carlsbad, CA, USA). Other solvents and reagents were used as received.

### 2.2. Synthesis of PEGDA/AA NPs

The synthesis of PEGDA/AA NPs was accomplished by surfactant-free emulsion polymerization with different molar ratios of PEGDA and AA (0.5:1, 1:1, and 1:0.5) using a procedure modified from a previous report [25]. In brief, 100 mL of deionized water was placed in a clean round-bottomed flask and warmed to 70 °C while flushing with nitrogen. To the warmed water, V50 (0.2% *w*/*w*) was added and stirred for >20 min. Meanwhile, PEGDA, AA, and MBA (10 wt%) were mixed in 5 mL of ethyl acetate before being slowly added into the initiator solution. The mixture was then stirred for 18 h to complete the polymerization reaction. Consequently, trace reagents and unreacted monomers were removed by dialysis against deionized water using dialysis tubing (MWCO = 3500 Da). The dialysis medium was replaced every 6 h for 3 days. Then, water was removed by lyophilization to give the dry PEGDA/AA NPs.

### 2.3. Characterization of PEGDA/AA Nanoparticles

The proton nuclear magnetic resonance spectra of the synthesized NPs were recorded. The NPs were homogeneously dispersed in deuterated water (D_2_O) and analyzed on an AVANCE III HD (Bruker, Billerica, MA, USA, operated at 300 MHz at 25 °C). The chemical shifts are given as parts per million (ppm). Additionally, the NPs were analyzed on an attenuated total reflection Fourier-transformed infrared (ATR-FTIR) spectrometer (Nicolet iS5, Thermo Scientific, Waltham, MA, USA) with 16 spectra collected between 400–4000 cm^−1^ with a resolution of 4.00 cm^−1^ [25].

The determination of the particle sizes and zeta potential of the PEGDA/AA NPs was carried out by dynamic light scattering (DLS) using a Zetasizer Nano ZS (Malvern Instruments, Malvern, UK) at 25 °C. The NPs were dispersed in ultrapure water (1 mg/mL) and sonicated with a probe sonicator to disaggregate the particles. The dispersant was diluted with ultrapure water (1:99) and added to the zeta cell through a 0.45 µm syringe filter. Each colloidal sample was measured in triplicate.

The morphology of the PDGDA/AA NPs was observed by a transmission electron microscope (TEM) (JEOL JEM-1400Flash, Tokyo, Japan) with an 80 kV accelerating voltage. The NPs were suspended in water and stained with 1% uranium acetate on a support grid. Furthermore, the appearance of the NPs was also determined using scanning electron microscopy (SEM) (Tescan Mira 3, Brno-Kohoutovice, Czech Republic). The lyophilized NPs were coated with a gold layer before analysis.

### 2.4. Drug Loading

Dox was loaded into the PEGDA/AA NPs using the adsorption method. Briefly, 20 mg of lyophilized PEGDA/AA NPs was suspended in 10 mL of Dox dissolved in ultrapure water (weight ratios of NPs:Dox varied by slowly increasing the amount of Dox to find optimal drug-loading condition (1:0.5, 1:1, and 1:2)). Thereafter, the mixtures were shaken overnight using a rotary mixer. The Dox-NPs were then collected by centrifugation (14,800 rpm for 10 min). The supernatant was discarded, and the pellet was washed twice with ultrapure water. The dried Dox-NPs were collected after freeze-drying.

The content of Dox in the NPs was determined by adding 1 mg of Dox-NPs in 1 mL of 0.1 M HCl in ultrapure water (2:98) and ultrasonicated for 1 h to instantly and completely extract the drug from the NPs, which was quantified by high-performance liquid chromatography (HPLC). The chromatographic conditions were adapted from Dharmalingam et al. (2014), with slight modification with a mobile phase consisting of a mixture of acetonitrile and water (pH 3) (30:70) delivered at a flow rate of 1.0 mL/min. The C-18 column (250 × 4.6 mm, 5 µm) was used with its temperature maintained at 30 °C, and the drug was detected by a UV–Visible detector (480 nm) [26]. The loading capacity (LC) and the loading efficiency (%LE) were calculated using Equations (1) and (2), respectively.

(1) $$\mathrm{LC}=\frac{\mathrm{The}\;\mathrm{amount}\;\mathrm{of}\;\mathrm{Dox}\;\mathrm{quantified}\;(\mu g)}{\mathrm{The}\;\mathrm{amount}\;\mathrm{of}\;\mathrm{Dox}\text{-}\mathrm{NPs}\;(\mathrm{mg})}$$

(2) $$\%\mathrm{LE}=\;\frac{\mathrm{The}\;\mathrm{amount}\;\mathrm{of}\;\mathrm{Dox}\;\mathrm{quantified}\;(\mathrm{mg})}{\mathrm{The}\;\mathrm{amount}\;\mathrm{of}\;\mathrm{Dox}\;\mathrm{added}\;(\mathrm{mg})}\times 100$$

### 2.5. Drug Release

The release of Dox from the PEGDA/AA NPs was carried out by dialysis with the release characteristics studied for two release media; phosphate buffer saline (at pH 5.0 and pH 7.4). Firstly, 10 mg of the Dox-NPs and free Dox (with equivalent amount of Dox to the Dox-NPs) were dispersed in the release medium (1 mL) and placed in a dialysis bag (molecular weight cut-off = 6000–8000 Da). The bags were submerged in 25 mL of the release medium and agitated at 75 rpm in an incubator shaker controlling the temperature (37 °C). At each pre-defined time point, 1 mL of the release medium was sampled and replaced with fresh medium.

### 2.6. Biocompatibility and Cytotoxicity

The biocompatibility of the blank NPs on the model intestinal epithelial cells (Caco-2) was evaluated using an MTT assay. The cells were cultivated in DMEM supplemented with 10% FBS, 100 mM L-glutamine, 1% non-essential amino acids, 1% sodium pyruvate, and 0.1% penicillin-streptomycin. To the 96-well plates, the cells were seeded to a density of 10,000 cells/well and stored in a controlled environment (5% CO_2_, 95% air, 37 °C). Different concentrations (1–5000 μg/mL) of blank PEGDA/AA NPs were prepared in DMEM and used to treat the cells for 24 h. The samples were removed, and the cells were washed with sterile 1× PBS pH 7.4 before the MTT solution in DMEM (0.5 mg/mL) was replaced and further incubated for 3 h. The resultant formazan crystals were dissolved in dimethyl sulfoxide (DMSO), and the absorbance was measured on a microplate reader (Multimode Microplate Reader, VICTOR Nivo™, Perkin Elmer, MA, USA) at 550 nm. The percentage of cell viability was calculated relative to the untreated control cells.

The cytotoxicity of free Dox and Dox-NPs towards Caco-2 cells and human colorectal adenocarcinoma cell line (HT-29) were also assessed using an MTT assay. The HT-29 cells were grown in DMEM with 10% FBS, 100 mM L-glutamine, 1% non-essential amino acids, and 1% penicillin-streptomycin. Free Dox and Dox-NPs (at a concentration range between 0.5–100 µg/ mL in serum-free DMEM) were used to treat both cell lines for 24 h. The relative cell viability was calculated, and the IC50 values were computed from a dose–response curve of log(concentration) vs. relative cell viability using GraphPad Prism v.5.01.

### 2.7. Cellular Uptake

Flow cytometry analysis was performed to investigate the uptake of Dox-NPs into the HT29 cells. The HT29 cells were seeded into a 24-well plate to the density of 50,000 cells/well and incubated for 24 h. The cells were then treated with either a solution of Dox or Dox-NPs prepared in serum-free DMEM at the concentration equivalent to the IC50 value of free Dox. At the specified time points (1, 2, 4, and 8 h), the cells were washed with PBS and serum-free DMEM and detached with Accutase^®^. Once dispersed homogeneously, the cells were fixed with 4% formaldehyde and stored at 4 °C until analysis. The mean fluorescent intensity (MFI) of Dox inside the cells was analyzed using an Attune^®^ NxT Flow Cytometer.

### 2.8. Cell Death Assay

An apoptosis cell death assay for free Dox and Dox-NPs was carried out using a dual staining technique. Briefly, HT-29 cells were seeded in a 6-well plate to a density of 50,000 cells/well and incubated in a controlled environment until 60–70% confluency was achieved. The cells were then treated with free Dox and Dox-NPs at various concentrations (equivalent to 1, 3, and 5 μM of Dox). After 24 h, the cells were washed, detached, and incubated with 1× annexin binding buffer in the dark. Consequently, the cell suspensions were stained with annexin V Alexa Fluor™ 647 conjugate and 1 μM SYTOX™ Green. After 15 min incubation in the dark, the samples were analyzed by flow cytometry [27].

### 2.9. Statistical Analysis

All experiments were carried out in triplicate. The data are presented as mean ± standard deviation (SD). Statistical analysis was calculated using Excel 2019, where *p* < 0.05 indicates a significant difference.

## 3. Results and Discussions

### 3.1. Synthesis of PGGDA/AA NPs

**Figure 1:**
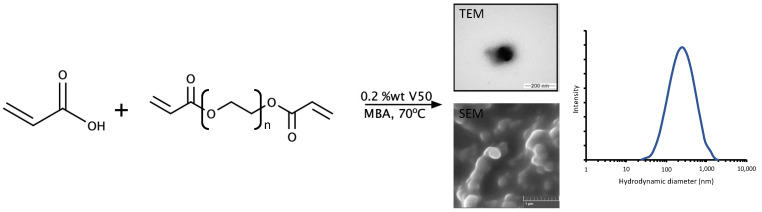
Synthesis of the of PEGDA/AA NPs with resulting NPs observed under TEM and SEM with corresponded particle size.

PEGDA/AA-based NPs were successfully synthesized by a surfactant-free emulsion polymerization reaction using the initiator V50, as shown in Figure 1.

**Figure 2:**
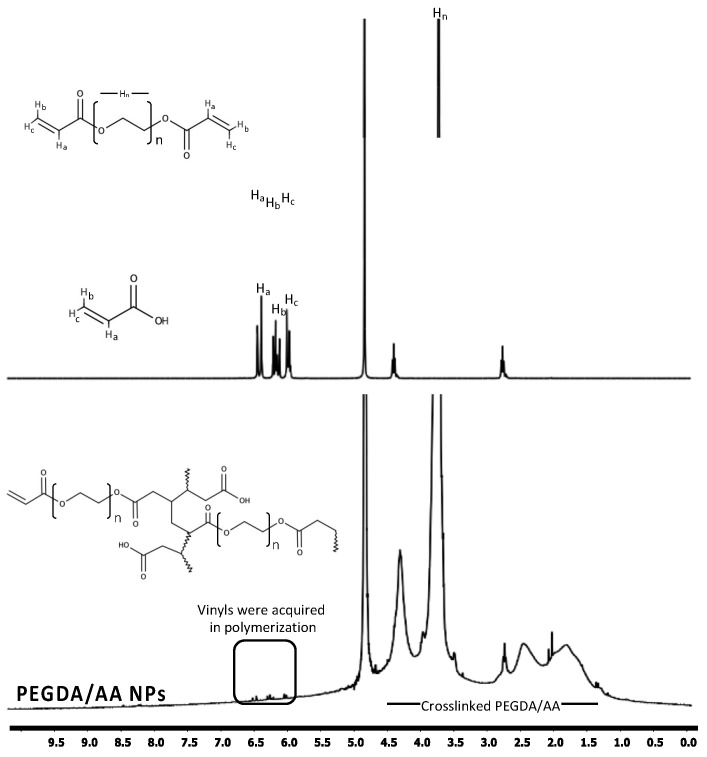
^1^H-NMR spectra of PEGDA, AA and PEGDA/AA NPs.

**Figure 3:**
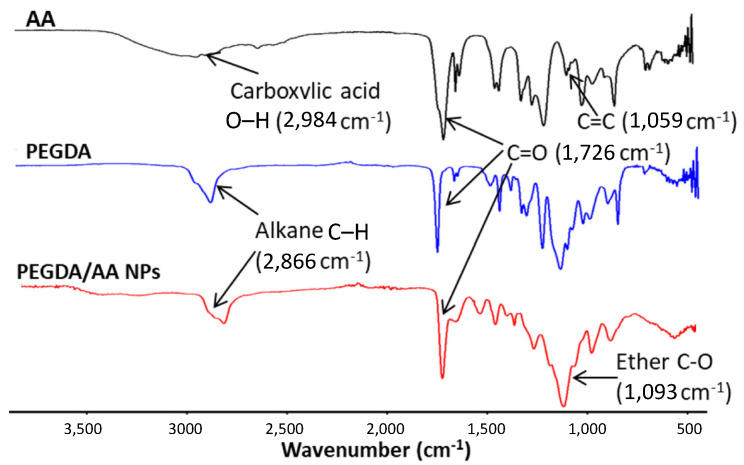
FTIR spectra of AA, PEGDA and PEGDA/AA NPs.

The structure of the NPs was examined by ^1^H-NMR and ATR-FTIR and was fully consistent with the proposed structure. The ^1^H-NMR spectra presented in Figure 2 illustrated that the signals appeared at 5.9, 6.2, and 6.6 ppm in the PEGDA spectrum were attributed to the vinyl groups of the molecule, whereas peaks at 2.7 and 4.4 ppm belonged to the alkyl chain and methylene groups adjacent to the ether group, respectively [28,29]. The vinyl group of AA was shown as doublets at 6.0, 6.3, and 6.7 ppm. However, the vinyl signals almost completely disappeared after the polymerization process, indicating the success in performing the reaction where the remaining peaks referred to the unreacted vinyl of PEGDA. The polymerized chain showed multiplet signals between 1.3–4.7 ppm, which represented the propagated polymer chain and PEG side chains [30]. The ATR-FTIR spectra of PEGDA/AA NPs are presented in Figure 3. The spectrum of PEGDA/AA NPs corresponded to the expected structure with O–H stretching of carboxylic acid from AA, C=O stretching which occurred in both monomers at 1726 cm^−1^, and asymmetrical and symmetrical bending vibrations of C–H alkane from the polymerized chain and PEGDA alkyl chain at 2866 cm^−1^ [31]. Moreover, the band at 1093 cm^−1^ represents the C–O stretching of PEGDA ether [30]. Considering the spectroscopic analyses, the PEGDA/AA NPs were successfully synthesized through the described procedure.

### 3.2. Particle Size, Size Distribution, Zeta Potential, and Morphology

The size, polydispersity index (PDI), and zeta potential of the PEGDA/AA NPs with different ratios of PEGDA:AA are given in Table 1. The result revealed that all the NPs were approximately 250 nm in diameter with a negative zeta potential due to the existence of the carboxylic acid group of AA. The variation of the NPs composition did not affect the average hydrodynamic diameter of the NPs but impacted the size distribution and the zeta potential. According to the examination, the imbalanced molar ratio of the monomers for this reaction (R1 and R3) led to a higher size distribution, which may affect the delivery efficiency of the drug to the target cell [32,33]. Additionally, the alteration of monomers ratio may affect the PDI, because the excessed monomer from the equivalence could gather and form NPs of homo-monomer, creating NPs of different construction and size. The reduction of acrylic acid in the reaction (R3) altered the zeta potential, which may lead to a lesser colloidal stability [34]. The PEGDA and AA ratio of 1:1 (R1) displayed the narrowest size distribution (*p* < 0.05), while maintaining a zeta potential value that promised good colloidal stability. As the NPs were intended to target passively, homogenous size distribution is an important criterion that needs to be considered [35], and therefore, this ratio was selected for further experiments.

The physical appearance of the NPs as observed under TEM and SEM (Figure 1) showed that the PEGDA/AA NPs had a spherical-like shape with particle size comparable to that obtained by DLS. The TEM images showed dispersion of the NPs with slight aggregation on the copper grid, whereas NPs agglomeration was observed under SEM (presumably because the sample was observed in a dried state with aggregation occurring upon lyophilization due to the hydrophilic nature and large surface area to volume ratio of the NPs) [36,37].

### 3.3. Drug Loading

**Figure 4:**
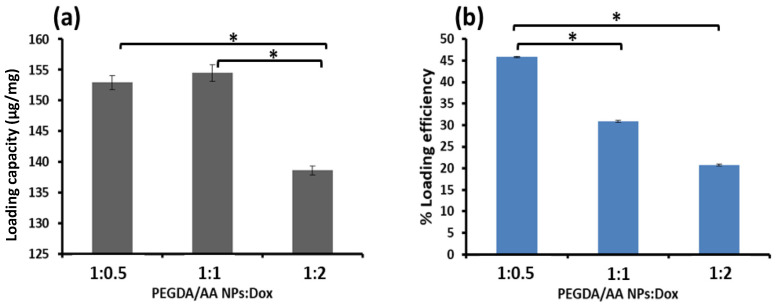
The (**a**) LC and (**b**) %LE of the Dox-loaded PEGDA/AA NPs. (* Significant difference at 95% CI) The drug experiments and Dox quantification were performed in triplicate. Each column represents the mean LC and %LE with standard deviation labelled.

To investigate the LC and %LE of Dox on the PEGDA/AA NPs, various weight ratios of Dox to NPs were loaded by the adsorption technique. The results are shown in Figure 4, where it can be observed that once a larger amount of Dox was added, the LC and %LE reduced. This determined that the Dox loading of the NPs was maximized at a PEGDA/AA NPs:Dox ratio of 1:0.5. The LC obtained was 152.0 ± 0.77 µg/ mg. A reduction in %LE upon higher Dox content confirmed that the NPs were fully loaded and that excess Dox was removed (Dox was presumably adsorbed onto the NPs by electrostatic interactions with the carboxylic groups of the NPs because Dox contains a protonated amino group under physiological conditions [38]).

### 3.4. In Vitro Drug Release

**Figure 5:**
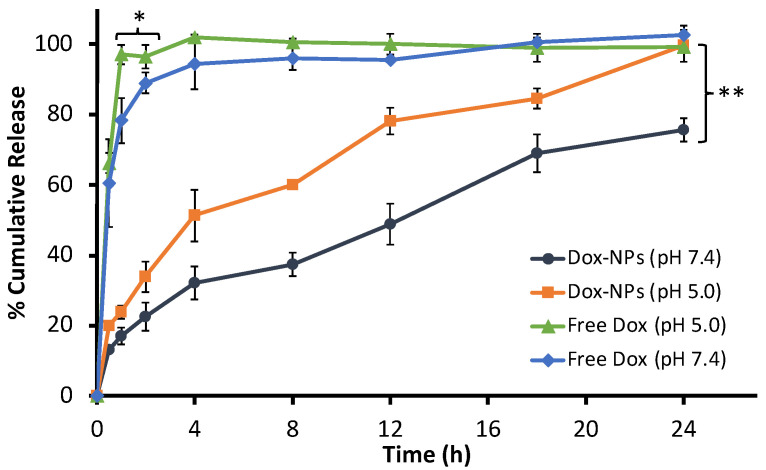
The release profiles of (–Δ–) Free Dox in pH 5.0, (–♦–) Free Dox in pH 7.4, (–∎–) Dox-NPs in pH 5.0, and (–●–) Dox-NPs in pH 7.4. (* Significant difference from free Dox pH 7.4 at 95% CI, ** Significant difference from Dox-NPs pH 7.4 at 95% CI).

The drug release behavior of Dox from the NPs was investigated by the dialysis method in PBS at two different pH values, which imitated the environments in the bloodstream (pH 7.4) and tumor (pH 5.0). Dox release is shown in Figure 5, which shows that free Dox was released rapidly from the dialysis tube at pH 5.0 and reached completion within an hour. At pH 7.4, the free Dox showed a slower release with 60% after 30 min and 100% after 4 h. This may be due to the hydrophilic properties and ionization properties of Dox, leading to rapid release with notable dissimilarity in a different environment [27]. At pH 5.0, Dox was released from the Dox-NPs at a slower pace than the free Dox with only 54% released in 4 h. The release from the Dox-NPs was slower at pH 7.4 with less than 50% of the Dox released after 12 h. Drug released was complete in 24 h for pH 5.0, presumably resulting from protonation of the carboxylate ions within the NPs under more acidic conditions, which resulted in the greater dissolution of the Dox [39]. The release profile of Dox clearly presented that the drug released from the NPs significantly slower at the physiological condition (pH 7.4) at all time points. Dox can freely diffuse across the dialysis upon protonation of the carboxylate groups, the dissociation of Dox from the NPs, and its hydrophilicity, which influenced the release. It seems that some Dox was not completely protonated at pH 7.4, causing the drug to be trapped within the NPs. However, the release was still gradually increased by time. These factors were related to the release profile of Dox-NPs obtained [40,41]. According to the literature review on Dox delivery to colorectal cancer, similar findings were found; for example, Lui et al. (2021) as well as Abedi et al. (2021) reported that the release of Dox was pH dependent, where a more rapid release was found at lower pH value, regardless of the NPs’ construction differences [42,43]. Furthermore, the release behavior of Dox from different kinds of NPs was found to collate to our findings. Norouzi et al. (2020) also reported faster release of Dox from iron oxide NPS in the tumor microenvironment (pH 5.0) compared to the physiological condition [44]. It can be suggested that passive targeting drug delivery requires the drug to reside in the carrier and be released upon reaching the target area. Therefore, the reduced Dox release under the physiological conditions was beneficial for Dox delivery, while the drug was expected to be released at the tumor site (more acidic pH) [39].

### 3.5. Cytotoxicity Evaluation

The biocompatibility of the blank NPs was evaluated on the Caco-2 cells, a model for the intestinal epithelial lining. The results are displayed in Figure S1 (ESI†). After treating the cells with the blank NPs at concentrations ranging from 1–5000 µg/mL, cell viability was over 85% for all concentrations, showing that the blank NPs were non-toxic to the intestinal epithelial cells, presumably related to the biocompatibility and safety of PEG and PEGDA [23,37].

The cytotoxicity of Dox and Dox-NPs toward the colorectal cancer cell line (HT-29) and Caco-2 cell was studied, and the results are shown in Figures S2 and S3 (ESI†), respectively. On HT-29 cells, free Dox had a highly toxic effect with an IC50 of 4.3 µg/mL, while the IC50 value of the DOX-NPs was 11.8 µg/mL. This may be due to direct penetration of the free drug into the nucleus, while the Dox from the NPs was gradually released. Both free Dox and Dox-NPs had similar effects on the Caco-2 cells. The experiment revealed that the Caco-2 cell viability of Dox and the Dox-NPs was greater than that found with the HT-29 cells (since the mechanism of action of Dox includes DNA damage and inhibition of cell proliferation, and the doubling time of Caco-2 (62 h) is greater than that of HT-29 (23 h), this leads to a lower level of death at the times analyzed).

### 3.6. Cellular Uptake

**Figure 6:**
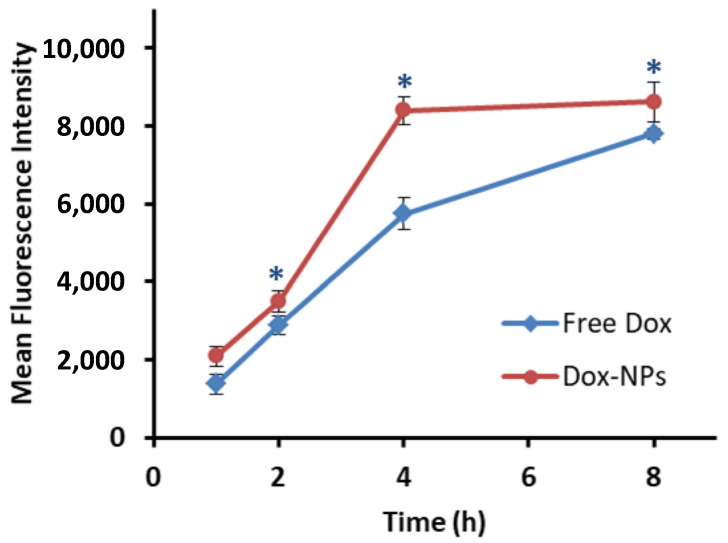
The cellular uptake of Dox and Dox-NPs into HT-29 cells at different time points (* Significant difference at 95% CI). The MFI was calculated from 10,000 events analyzed with the laser line of 488 nm by flow cytometer.

Cellular internalization is a crucial parameter to demonstrate the effectiveness of a drug-loaded nanocarrier preparation. The cellular uptake results from flow cytometry analysis are displayed in Figure 6. Over 2 h to 8 h, the MFI of Dox-NPs was significantly higher than that of free Dox (*p* < 0.05), indicating a higher content of Dox inside the colorectal cancer cells. Free Dox could penetrate the cells and rapidly interfere with cell proliferation, causing rapid cell death. Meanwhile, the Dox-NPs were taken up by the cell in the form of a drug-loaded nanocarrier and accumulated within the cell led to a higher MFI and slowly released the drug to perform its action. The slow release of the drug from the nanocarrier resulted in higher IC50 values of Dox-NPs [45]. The cellular uptake behavior of NPs likely occurred via endocytosis, while the free Dox, which is a water-soluble molecule, was internalized into the tumor cell via passive diffusion [46].

### 3.7. Apoptosis

**Figure 7:**
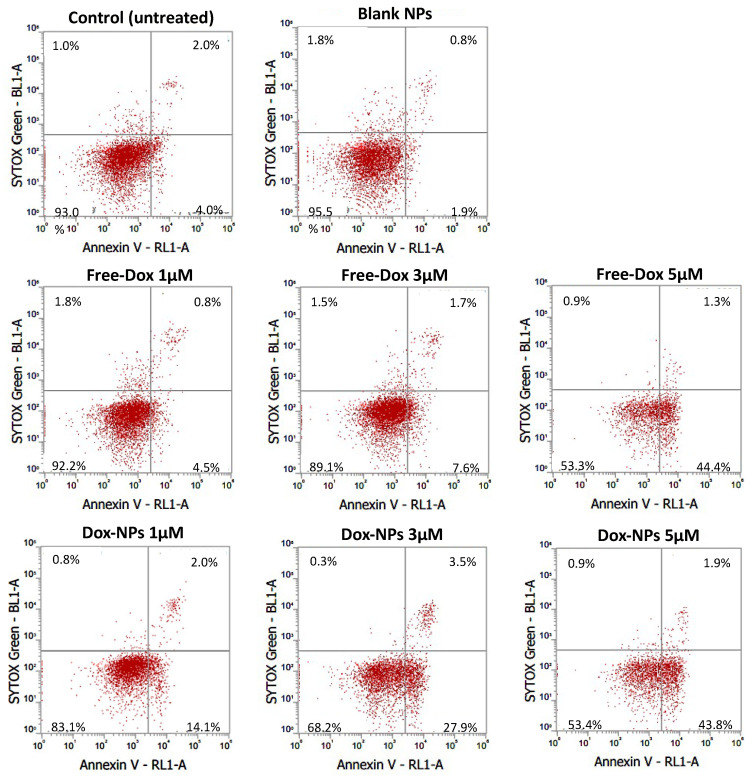
Apoptosis assay of HT-29 cells after treating with control (untreated), blank NPs, free Dox (1, 3, and 5 µM), and Dox-NPs (1, 3, and 5 µM). The experiments were performed using double staining technique of annexin Alexa Fluor^TM^ 647 conjugate and SYTOX^TM^ Green. The analysis was acquired from 10,000 events.

A double-staining method was performed to assess the HT-29 cell death pathway and analyzed via flow cytometry. The assay identified live, early apoptotic, late apoptotic, and necrotic cells upon cells staining with an annexin V Alexa Fluor™ 647 conjugate and/or SYTOX™ Green. The results are shown in Figure 7. Over 90% of the untreated control cells and those treated with blank NPs were live and showed no cytotoxic effects. Free Dox at lower concentrations did not show significant cell death but the effect was greatly enhanced once the concentration became higher. This suggested that the concentration of free Dox must reach an effective concentration to appropriately enter the cell membrane and nucleus, perhaps saturating albumin binding. However, Dox-NPs induced apoptosis in HT-29 cells in a dose-dependent manner. At 1 µM and 3 µM, DOX-NPs showed higher apoptosis cell death (16% and 31%, respectively) than free Dox (5% and 9%, respectively). The nanocarrier perhaps played a role in drug internalization and drug release within the cell. Therefore, the cells were affected by the drug at lower concentrations. At a concentration of 5 µM, apoptosis-directed cell death was approximately the same (~46%), showing that an effective “killing” concentration to HT-29 cells was reached. Note that an apoptosis cell death route is desirable, as this process prevents plasma membrane rupture and the leakage of cellular materials, leading to a less inflammatory type death [27]. This also aligned with previous in vitro studies that suggested that NP drug conjugates induced stronger activation of apoptosis [18].

## 4. Conclusions

PEGDA/AA NPs were synthesized as nanocarriers for Dox to allow delivery into colorectal cancer cells. The NPs were spherical, nicely mono-sized, and a had negative surface charge (via zeta analysis). Dox was adsorbed onto/into the NPs via electrostatic interactions. The release of Dox was sustained once incorporated onto the PEGDA/AA NPs, with the drug releasing more rapidly in an acidic environment (akin to that around a tumor) than under normal physiological conditions. The synthesized NPs were biocompatible with the intestinal epithelial lining. Dox-NPs were able to kill the cancer cell with an acceptable IC50, and accumulated in the cell and acted against cancer via apoptosis, a killing mechanism that is non-inflammatory compared to free Dox. In summary, the synthesized PEGDA/AA NPs could be a tool for chemotherapeutic drug delivery to colon cancer cells, and in vivo experiments are warranted.

## Figures and Tables

**Table 1:** Particle size, PDI, and zeta potential of the PEGDA/AA NPs. (* Significant difference from PEGDA:AA (0.5:1)), *n* = 3.

Reaction	PEGDA:AA	Particle Size (nm)	PDI	Zeta Potential (mV)
R1	0.5:1	247.6 ± 4.0	0.40 ± 0.02	−29.9 ± 0.5
R2	1:1	232.0 ± 4.9 *	0.25 ± 0.03 *	−18.0 ± 0.1 *
R3	1:0.5	254.0 ± 7.5	0.39 ± 0.02	−8.0 ± 0.6 *

## Data Availability

Not applicable.

## Supplementary Materials

The following supporting information can be downloaded at: https://www.mdpi.com/article/10.3390/pharmaceutics14030479/s1, Figure S1: Biocompatibility study of blank NPs on Caco-2 cells; Figure S2: Percentage of cell viability of HT-29 cells after treatment with free Dox and Dox-NPs; Figure S3: Percentage of cell viability of Caco-2 cells after treatment with free Dox and Dox-NPs.
