# Supplementary material for: Synthesis of Polyethylene Glycol Diacrylate/Acrylic Acid Nanoparticles as Nanocarriers for the Controlled Delivery of Doxorubicin to Colorectal Cancer Cells
Source: Pharmaceutics. 2022 Feb 22;14(3):479. doi: 10.3390/pharmaceutics14030479 (PMC8950920; doi:10.3390/pharmaceutics14030479)
Supplement: Supplementary file 1 [file pharmaceutics-14-00479-s001.zip › pharmaceutics-1602685-supplementary.pdf]

Electronic supplementary information

Synthesis of polyethylene glycol diacrylate/acrylic acid nanoparticles as nanocarriers for the controlled delivery of doxorubicin to colorectal cancer cells

Yin Yin Myat [a], Tanasait Ngawhirunpat [a], Theerasak Rojanarata [a], Praneet Opanasopit [a], Mark Bradley[b], Prasopchai Patrojanasophon [a], and Chaiyakarn Pornpitchanarong [a,*]

[a] Pharmaceutical Development of Green Innovations Group (PDGIG), Faculty of Pharmacy, Silpakorn University, Nakhon Pathom, 73000, Thailand

[b] School of Chemistry, University of Edinburgh, David Brewster Road, Edinburgh, EH9 3FJ, United Kingdom

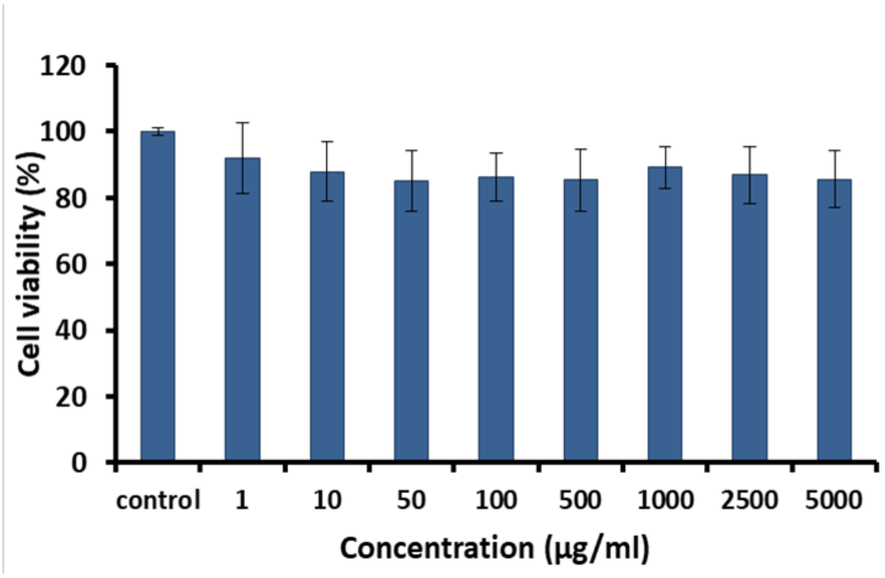

Figure S1. Biocompatibility study of blank NPs on Caco-2 cells.

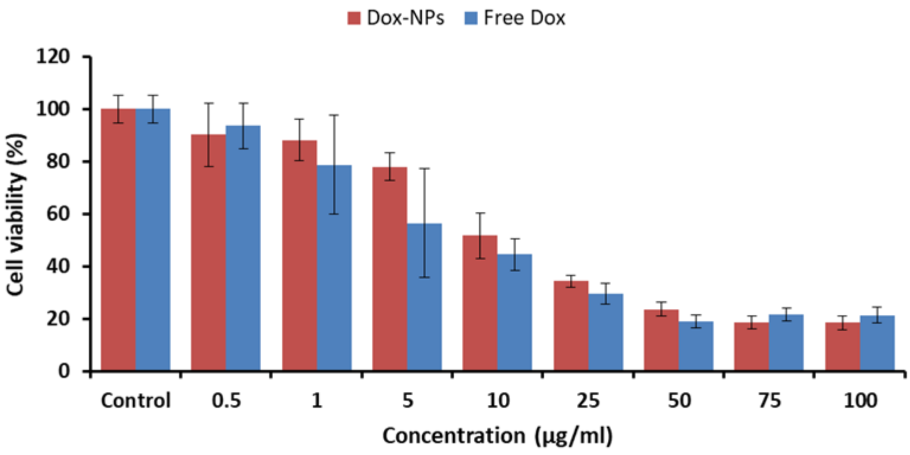

Figure S2. Percentage of cell viability of HT-29 cells after treatment with free Dox and Dox-NPs.

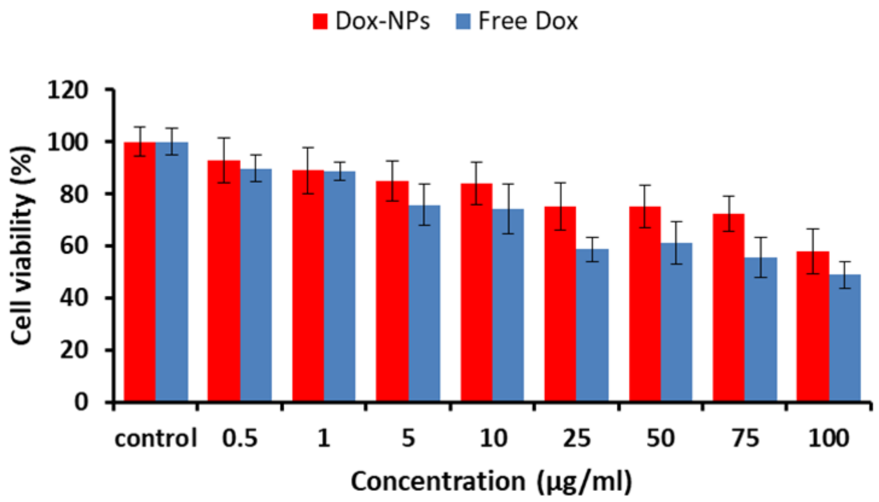

Figure S3. Percentage of cell viability of Caco-2 cells after treatment with free Dox and Dox-NPs.
